# Is Fatty Liver Associated With Depression? A Meta-Analysis and Systematic Review on the Prevalence, Risk Factors, and Outcomes of Depression and Non-alcoholic Fatty Liver Disease

**DOI:** 10.3389/fmed.2021.691696

**Published:** 2021-06-30

**Authors:** Jieling Xiao, Lincoln Kai En Lim, Cheng Han Ng, Darren Jun Hao Tan, Wen Hui Lim, Cyrus S. H. Ho, Eunice Xiang Xuan Tan, Arun J. Sanyal, Mark D. Muthiah

**Affiliations:** ^1^Yong Loo Lin School of Medicine, National University of Singapore, Singapore, Singapore; ^2^Department of Psychological Medicine, Yong Loo Lin School of Medicine, National University of Singapore, Singapore, Singapore; ^3^National University Centre for Organ Transplantation, National University Health System, Singapore, Singapore; ^4^Division of Gastroenterology and Hepatology, Department of Medicine, National University Hospital, Singapore, Singapore; ^5^Division of Gastroenterology, Hepatology, and Nutrition, Department of Internal Medicine, Virginia Commonwealth University, Richmond, VA, United States

**Keywords:** mood disorder, metabolic disease, fatty liver, evidence-based practice, mental health

## Abstract

**Background and Aims:** Both non-alcoholic fatty liver disease (NAFLD) and depression have a high global prevalence which is projected to increase further. While studies exploring the association have been done, there are conflicting data. This study aims to assess the prevalence and association between depression and NAFLD.

**Methods:** Medline and Embase were searched from inception to March 3, 2020. Meta-analysis of proportions using the generalized linear mix model was conducted to analyze the pooled prevalence of depression in NAFLD patients. Risk factors for depression in NAFLD patients were evaluated in conventional pairwise meta-analysis.

**Results:** Ten studies involving 2,041,752 NAFLD patients were included. Pooled prevalence of depression was 18.21% (CI: 11.12–28.38%) in patients with NAFLD and 40.68% (CI: 25.11–58.37%) in patients with non-alcoholic steatohepatitis (NASH). NAFLD resulted in significantly higher risk of development of depression (OR: 1.29, CI: 1.02–1.64, *p* = 0.03). NASH patients had a significantly higher risk of depression compared with NAFLD patients (RR: 2.83, CI: 2.41–3.32, *p* < 0.001). Diabetes, body mass index (BMI), female sex, smoking, and history of pulmonary disease were significant risk factors for depression in NAFLD patients.

**Conclusion:** This study demonstrated a high prevalence of depression in NAFLD patients and a significant association between both conditions. Furthermore, patients with NASH had a significantly higher risk of depression compared with those with NAFLD. Diabetes, BMI, history of lung disease or smoking, and female gender were significant risk factors. Further studies investigating the pathophysiological mechanism underlying depression and NAFLD are needed.

## Introduction

Non-alcoholic fatty liver disease (NAFLD) is the most common cause of chronic liver disease, with an estimated global prevalence of 25%. This is further projected to increase in conjunction with the rising rates of metabolic syndrome and obesity (1–4). However, the influence of depression on NAFLD has yet to be well-described. The Global Burden of Disease study from 1990 to 2017 reported a 49.86% increase in the incidence of depression (5), with at least one in five people experiencing depression in their lifetime (6–8). Depression is often comorbid of many chronic diseases and incrementally worsens health outcomes (9, 10). Multiple community- and population-based studies have reported that patients with depression have a 2-fold increased risk of developing metabolic syndrome (11–13). Depression is also highly prevalent in the diabetic population, affecting more than one-quarter of both type 1 and type 2 diabetics (14). A meta-analysis of longitudinal studies also confirmed the reciprocal relationship between obesity and depression (15) and increased cardiac mortality in patients with this comorbidity (16).

While studies have been conducted to describe the relationship between depression and NAFLD, previous literature has reported conflicting results, varying from a strong association (17, 18) to no association (19, 20). A study involving a database of 567 patients with biopsy-proven NAFLD estimated that 67.5% of patients had depressive symptoms and showed that they were associated with histological severity of NAFLD (21). It is unclear if depression affects NAFLD due to underlying risk factors or if depression is independently associated with NAFLD (22). Thus, this meta-analysis and systematic review aims to assess the prevalence, associations, risk factors, and outcomes between depression and NAFLD.

## Methods

### Search Strategy

This review was synthesized with reference to the Preferred Reporting Items for Systematic Reviews and Meta-Analyses (PRSIMA) guidelines (23). A search was conducted on Medline and Embase databases to identify relevant articles from inception up to March 3, 2020. Keywords and MeSH terms synonymous to “NAFLD” and “depression” were applied in the search strategy to identify relevant articles. The full search used was as follows: (depress^*^.tw. or exp Depression/or MDD.tw.) AND (NAFLD or NASH or ((liver or hepatic) AND (fatty or steatosis or steatoses))).tw. or (exp Fatty Liver/or steatohepat^*^tw.). In addition, a sieve was conducted on the references of included articles. Abstracts were imported into EndNote X9 for removal of duplicates and for the initial sieve.

### Study Selection and Data Extraction

Two authors (XJL and LLKE) were involved in the screening of abstracts to check the eligibility for inclusion, with disputes being resolved through consensus from a third independent author. Retrospective and prospective cohort studies, case–control, and cross-sectional studies were considered for inclusion, while editorials, systematic reviews, meta-analyses, and commentaries were excluded. Additionally, only English language articles were considered for inclusion. Studies were included according to the following criteria: i) studies regarding the prevalence, risk factors, and outcomes of depression and ii) studies where patients had a diagnosis of NAFLD made *via* liver biopsy, imaging techniques (radiologic testing and abdominal ultrasound), or International Classification of Diseases. Studies relating to the diagnosis of non-alcoholic steatohepatitis (NASH) by self-reported physician diagnosis were also included. Since NASH is a subgroup of NAFLD, a definitive diagnosis of NASH would equate to the presence of NAFLD. As with a previous article, the diagnosis of depression was subclassified into self-reported, self-rated, and clinician-rated (24). Self-reported diagnosis includes identification of depression through self-reporting of medical history, while self-rated diagnosis of depression comprises of patient responses from the Patient Health Questionnaire-9 (PHQ-9), Hospital Anxiety and Depression Scale (HADS), Korean Center for Epidemiological Studies-Depression Scale (CES-D), and Beck's Depression Inventory scale. Clinician-rated diagnosis comprises of depression diagnosed by a psychiatrist according to the criteria of the Diagnostic and Statistical Manual of Mental Disorders, 4th edition, text revision (DSM-IV-TR) and International Classification of Disease, Ninth Revision (ICD-9) and Tenth Revision (ICD-10) codes. Pediatrics studies were excluded from the analysis.

Relevant data from each article were extracted by a pair of independent authors onto a structured proforma. Baseline demographics, including but not limited to author, year, sample size, country, mean age, percentage of diabetes, and prevalence of depression, were extracted. The main outcomes of interest were the prevalence of depression in NAFLD and NASH patients and risk factors including gender, diabetes mellitus, body mass index (BMI), hypertension, hyperlipidemia, history of smoking, history of lung disease, history of cancer, history of heart diseases, history of neurologic diseases, and race.

### Statistical Analysis and Quality Assessment

All analysis was done in R studio (Version 1.3.1093) and RevMan 5.4. Statistical significance was considered for outcomes with a *p*-value < 0.05. A single-arm analysis of binary outcomes was pooled in the form of proportions using the generalized linear mixed model (GLMM) with Clopper–Pearson intervals to stabilize the variance (25, 26). Simulation studies have found that the GLLM model provides the most accurate estimate in single-arm meta-analysis (25). A sensitivity analysis was done to include only studies from biopsy-proven NAFLD. Next, a subgroup analysis was conducted to account for the differences in the rate of depression between clinician-diagnosed, self-reported, and self-rated diagnosis. Based on the pooled proportions of single-arm studies, the respective relative risks (RR) of depression in NASH (*p*_1_) vs. NAFLD (*p*_2_) patients was calculated as the ratio of the pooled proportions $\frac{p_{1}}{p_{2}}$ of patients with depression in each subgroup (27). The lower (*LCL*) and upper (*UCL*) bounds for the 95% confidence intervals were estimated using the Katz-logarithmic method, wherein *n*_1_ and *n*_2_ represent the respective number of patients receiving transplanted allografts fixated using each of the three methods. Additionally, the *p*-value was calculated after a natural log transformation of the relative risk *z*-score (28).

$$\begin{array}{l} LCL=RRe^{\left(-1.96\;\times \sqrt{\frac{1-p_{1}}{n_{1}p_{1}}+\frac{1-p_{2}}{n_{2}p_{2}}}\right)} \end{array}$$

$$\begin{array}{l} UCL=RRe^{\left(1.96\;\times \sqrt{\frac{1-p_{1}}{n_{1}p_{1}}+\frac{1-p_{2}}{n_{2}p_{2}}}\right)} \end{array}$$

$$\begin{array}{l} p-value=e^{\left(-0.717\;\times \;\left|\frac{\ln RR}{\frac{\ln UCL-\;\ln LCL}{2\;\times \;1.96}}\right|-0.416\;\times \;{\left(\frac{\ln RR}{\frac{\ln UCL-\;\ln LCL}{2\;\times \;1.96}}\right)}^{2}\right)} \end{array}$$

In the analysis of risk factors, a conventional pairwise analysis was done using odds ratios (OR) and mean difference (MD) with the Mantel–Haenszel and inverse variance, respectively (29, 30). For outcomes with insufficient articles for a meta-analysis, a systematic synthesis of literature was used to represent available data. Statistical heterogeneity was assessed *via I*^2^ and Cochran *Q* test values, where an *I*^2^ value of 0 to 40% indicates low heterogeneity, while values of 30–60, 50–90, and 75–100% were classified as moderate, substantial, and considerable heterogeneity, respectively (29, 31). A Cochran *Q* test with *p*-value of ≤ 0.10 was considered significant for heterogeneity. A random effects model was used in all analysis regardless of heterogeneity as recent evidence suggests that it provides more robust outcome measures compared with the alternative fixed effects models. Publication bias was not assessed with the lack of a suitable tool in single-arm meta-analysis to assess publication bias and small quantity of included studies (32, 33). Quality assessment of the included articles was done with the Joanna Briggs Institute (JBI) Critical Appraisal Tool (34). The JBI assessment rates the risk of bias of cohort studies on the premises of appropriateness of sample frame, sampling method, adequacy of sample size, data analysis, methods for identification and measurement of relevant conditions, statistical analysis, and response rate adequacy and is the most widely used tool in prevalence meta-analysis.

## Results

### Summary of Included Articles

**Figure 1:**
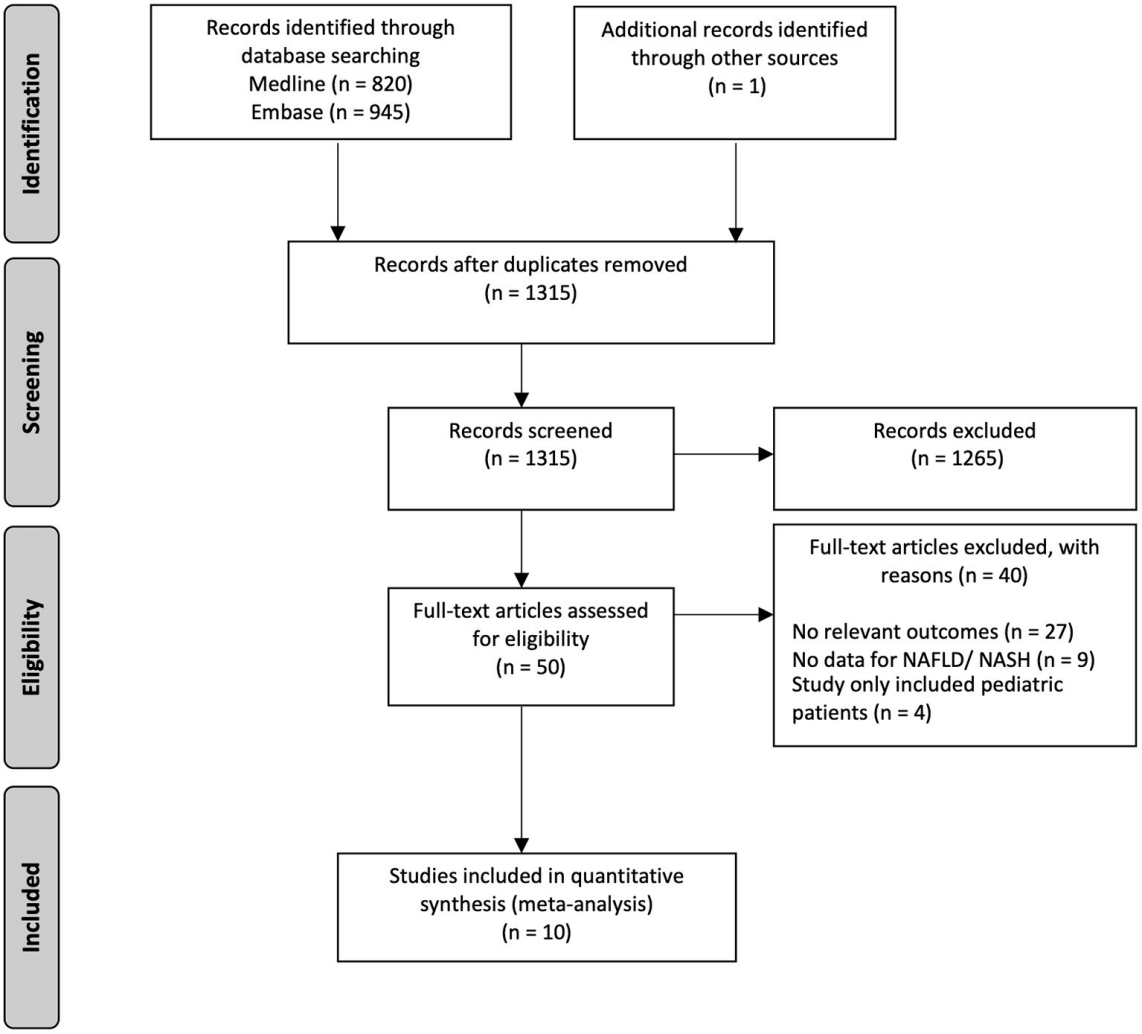
PRISMA flowchart of the included studies.

In the initial search strategy, 1,766 references were identified, of which 1,315 references were screened after the removal of duplicates. After initial screening, a full-text review was done for 50 articles, and finally, 10 articles involving 2,041,752 NAFLD patients were included in the analysis (Figure 1). Of the included studies, one is a prospective study (35), while the remainder are retrospective studies (17, 21, 22, 36–41). Depression was identified through clinician-rated scales [National Institute of Mental Health Diagnostic Interview Schedule (DIS), Version III (36); DSM-IV-TR (35) and International Classification of Disease, Ninth Revision (ICD-9) and Tenth Revision (ICD-10) (41, 42)], self-rated scales [HADS (21), CES-D (38), Beck Depression Inventory scale (22)], and self-reported physician diagnosis (17, 37, 40). NAFLD was diagnosed through liver biopsy in four studies (17, 21, 35, 40) and imaging techniques in four studies (17, 22, 38, 40) and identified through ICD-9 and ICD-10 codes in two studies (41, 42). NASH was diagnosed through liver biopsy in one study (36) and identified through self-reported physician diagnosis in another study (37). A summary of the included articles and quality assessment can be found in Supplementary Tables 1, 2, respectively.

### Development of Depression in NAFLD

**Figure 2:**
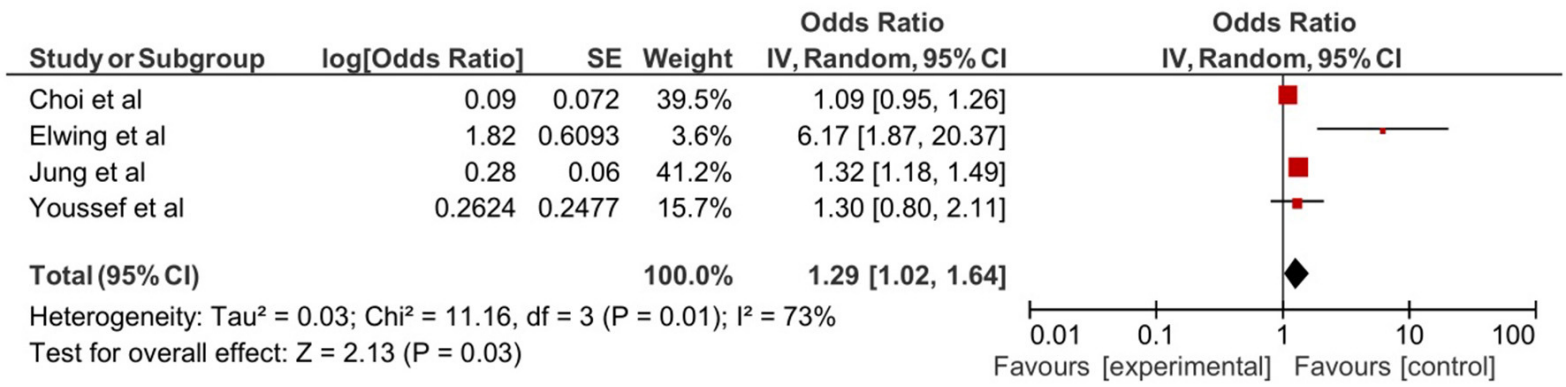
Forest plot of depression in non-alcoholic fatty liver disease (NAFLD).

Five studies assessed the association of depression with NAFLD (21, 22, 36, 38, 42). In the pooled analysis of the four retrospective studies involving 38,407 patients, NAFLD resulted in a significant increase in the risk of depression (OR: 1.29, 95% CI: 1.02–1.64, *p* = 0.03, Figure 2). A retrospective follow-up study by Labenz et al. assessed the incidence of depression in 19,871 NAFLD patients over 10 years. The 10-year incidence of depression was 21.2% in patients with NAFLD compared with 18.2% of individuals without NAFLD. The hazard ratio was 1.21 (95% CI: 1.14–1.26, *p* < 0.001) risk increase in development depression in NALFD patients after adjusting for confounders including diabetes mellitus, cardiovascular diseases, asthma, chronic obstructive pulmonary disease (COPD), and cancer (42).

### Prevalence of Depression in NAFLD

**Figure 3:**
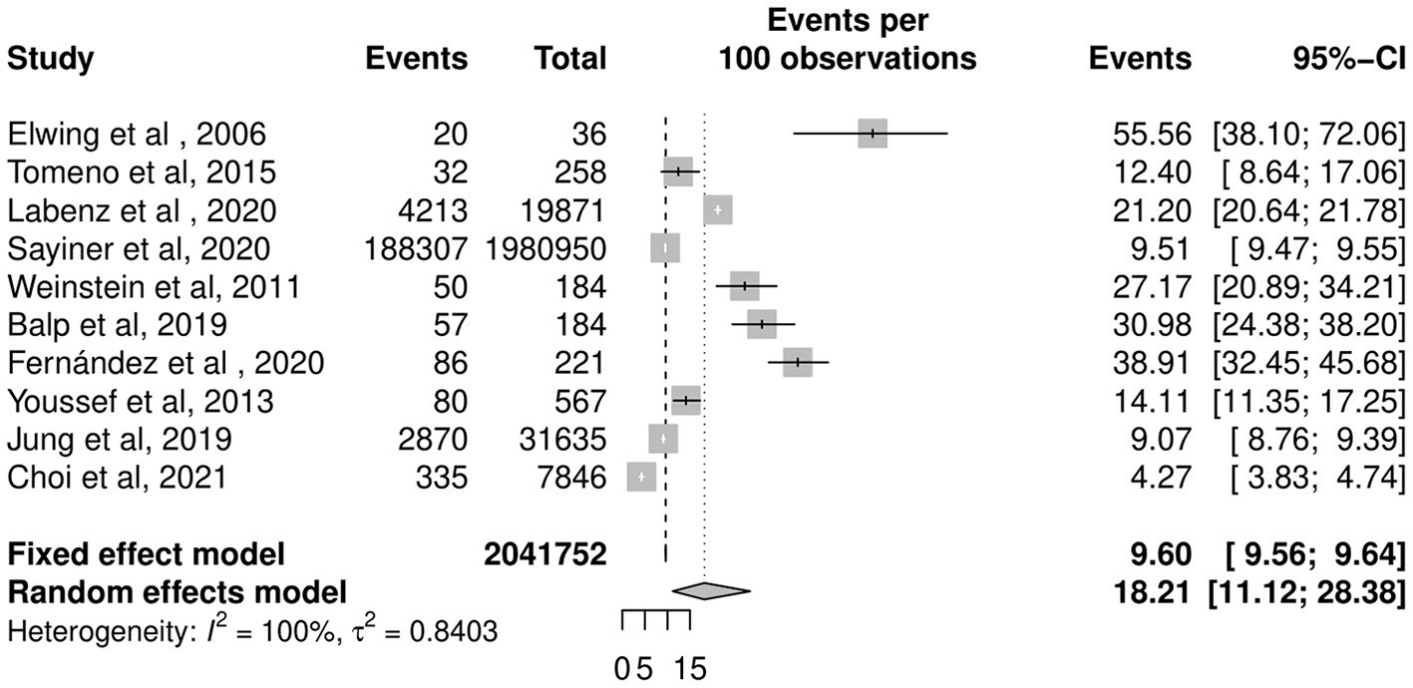
Pooled prevalence of depression in NAFLD patients.

The overall pooled prevalence of depression in patients with NAFLD was 18.21% (CI: 11.12–28.38%, Figure 3) in 2,041,752 individuals. The pooled prevalence of depression in biopsy-proven NAFLD was 22.68% (CI: 8.93–46.75%).

A subgroup analysis was conducted to compare between NASH and NAFLD. The prevalence of depression in NASH patients is 40.68% (CI: 25.11–58.37%), significantly higher than the pooled prevalence of depression in NAFLD patients at 14.39% (CI: 8.89–22.45%). Compared with patients with NAFLD, patients with NASH had a significantly higher risk of depression (RR: 2.83, CI: 2.41–3.32, *p* < 0.001).

To account for differences in the diagnosis of depression as a potential source for heterogeneity, estimates were stratified by diagnostic criteria of depression. Prevalence of depression in NAFLD patients in four studies where depression was identified through clinician-rated scales was 20.26% (CI: 9.38–38.42%) (35, 36, 41, 42), which was lower than self-reported diagnosis used in three studies where prevalence was 32.43% (CI: 27.03–38.33%) (17, 37, 40) and higher than self-rated depression reported in three studies where prevalence was 8.19% (CI: 4.66–14.01%) (21, 22, 38).

### Factors Associated With Depression in NAFLD

When comparisons were made between NAFLD patients with depression and NAFLD patients without depression, diabetes (OR: 1.71, CI: 1.15–2.55, *p* = 0.007), BMI (MD: 1.89, CI: 0.97–2.80, *p* < 0.0001), and female sex (OR: 0.57, CI: 0.35–0.93, *p* = 0.02) were significant risk factors associated with depression in NAFLD patients. However, hypertension (OR: 1.54, CI: 1.00–2.37, *p* = 0.05) and hyperlipidemia (OR: 1.18, CI: 0.61–2.28, *p* = 0.623) were not significant risk factors for depression in NAFLD patients.

In addition, Weinstein et al. (17) reported history of smoking and history of lung disease as statistically significant risk factors independently associated with depressions in NAFLD patients (OR: 4.132, CI: 1.224–13.95, *p* = 0.0096 and OR: 4.621, CI: 1.346–15.92, *p* = 0.0087, respectively). A previous history of cancer (OR: 1.370, CI: 0.394–4.765, *p* = 0.621), heart diseases (OR: 2.750, CI: 0.377–20.07, *p* = 0.319), or neurologic disease (OR: 2.787, CI: 0.544–14.29, *p* = 0.219) had no significant association with depression. African American descent was not associated with the development of depression (OR: 0.198, CI: 0.024–1.629, *p* = 0.147).

### Outcomes of NAFLD With Depression

Two studies, Tomeno et al. and Sayiner et al. reported on the outcomes of NAFLD patients comorbid with depression. Tomeno et al. compared the clinical response of 32 NAFLD patients with major depressive disorder (MDD) and 226 NAFLD patients without MDD after 48 weeks of standard care involving basic education, lifestyle change counseling, and medication control. Parameters used in the calculation of hepatic steatosis index and fatty liver index such as body weight, serum AST, ALT, and GGT levels were significantly improved in NAFLD patients without MDD after 48 weeks of standard care. In contrast, there were no statistically significant improvements in these parameters for NAFLD patients with MDD (35). Sayiner et al. analyzed depression as an independent predictor of overall 1-year mortality in NAFLD patients in both inpatient and outpatient settings. The odds for overall 1-year mortality of NAFLD patients with depression was statistically significant in both inpatient (OR: 1.07, CI: 1.05–1.09) and outpatient settings (OR: 1.21, CI: 1.18–1.25) (41).

## Discussion

**Figure 4:**
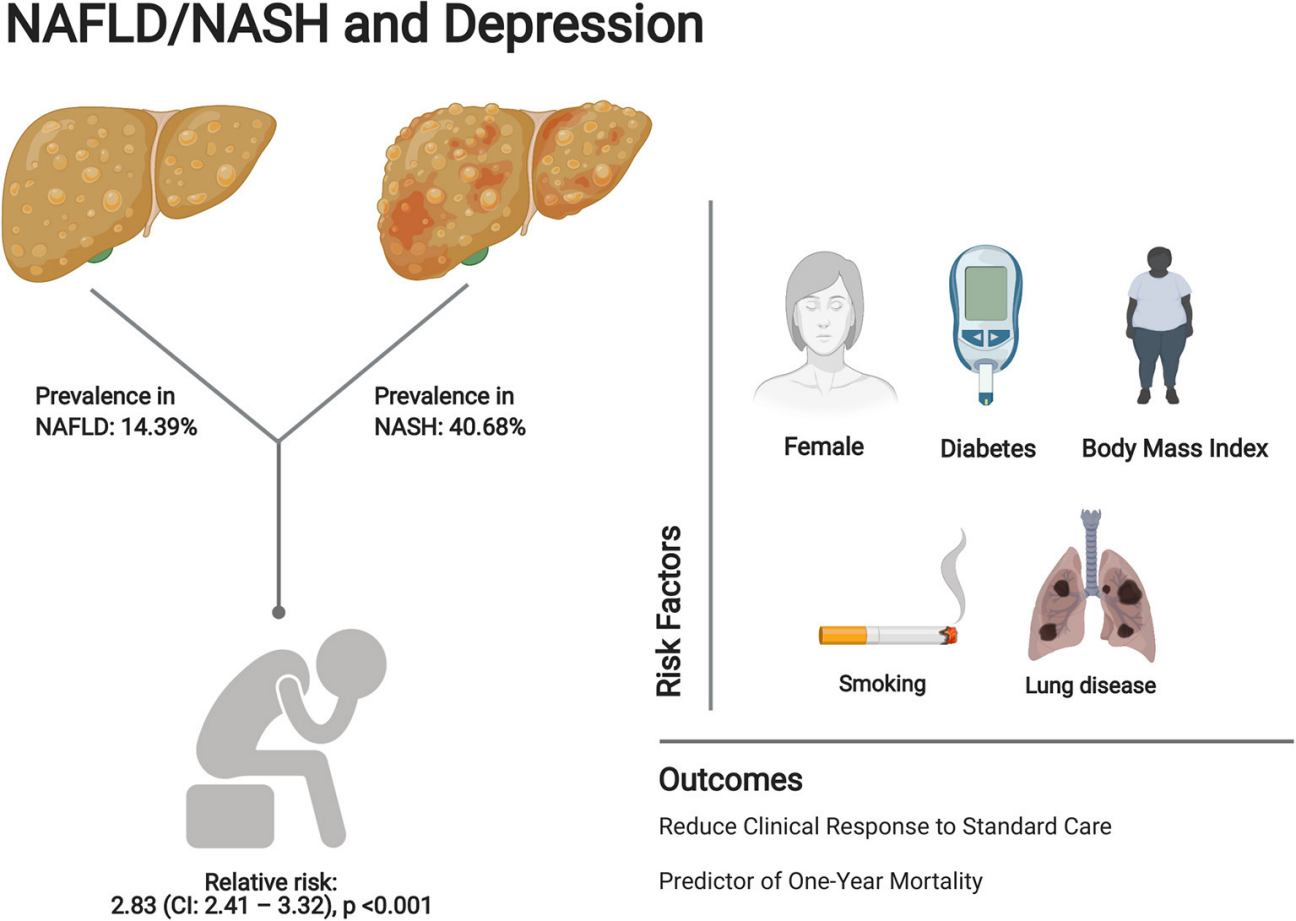
Summary of prevalence, risk factors, and clinical outcomes of depression in NAFLD patients. Image created with Biorender.com.

The projected rise in the incidence of NAFLD mirrors the obesity epidemic, which at present affects at least one-third of the world (43). This systematic review and meta-analysis reports 15.76% prevalence of depression in NAFLD patients. The risk of development of depression was significantly increased in patients with NAFLD (OR: 1.29, 95% CI: 1.02–1.64, *p* = 0.03). A 10-year follow-up by Labenz et al. also showed a significant association between depression and NAFLD (HR: 1.21, 95% CI: 1.14–1.26, *p* < 0.001). Female sex, diabetes, BMI, history of smoking, and history of lung disease were associated with the development of depression. The relative risk of depression between NASH and NAFLD patients was RR: 2.83 (CI: 2.41–3.32, *p* < 0.001, Figure 4). In truth, the actual prevalence of clinically diagnosed depression is likely to be higher than that reported, especially among Asians where traditional screening methods for depression have been reported to have a poorer detection rate (44).

The incidence of depression is likely to be higher in patients with NAFLD as compared with those without, as shown by Labenz et al. in their large population-based study (42). Importantly, the authors controlled for variables including diabetes and obesity, demonstrating increased incidence of depression in patients with NAFLD independent of these comorbidities. While the studies included in our manuscript are unable to show causality, there is emerging evidence, which may shed light on the associations between depression and NAFLD. Systemic inflammation plays a putative role in the pathogenesis of NAFLD and depression, and the progression of both diseases is seen in states of increased oxidative stress (45). Increased levels of proinflammatory cytokines such as interleukin-6, interleukin-1 beta, and tumor necrosis factor alpha may contribute to systemic inflammation leading to depression in NAFLD patients (42, 46, 47). Another plausible association between NAFLD and depression would be the role of serotonin in the pathogenesis of depression as there is increased expression of serotonin catalyzing enzymes in patients with NASH (22).

In our study, diabetes was a significant risk factor for the development of depression (OR: 1.71, CI: 1.15–2.55, *p* = 0.007). Egede et al. reported diabetic patients to be almost twice as likely to be diagnosed with depression compared with the general population (48). BMI was identified as a significant risk factor for the development of depression (MD: 1.89, CI: 0.97–2.80, *p* < 0.0001). There are also several studies reporting an increased prevalence of depression with higher BMI (49, 50). Luppino et al. in their meta-analysis of obesity and depression showed that obesity at baseline had increased the risk of onset of depression at follow-up, with OR 1.55 (95% CI 1.22–1.98, *p* < 0.001) (15).

The risk factors of depression in NAFLD patients were associated with increased systemic inflammation as observed in smokers and patients with chronic lung disease. Female gender was also associated with increased odds of depression in NAFLD patients. It is known that females are at higher risk of depression than males, and a study in 2015 by Moieni suggested that during an induced state of inflammation, women encountered greater increases in depression (51). Smoking has also been identified as a risk factor for depression, possibly due to the role of nicotine receptors as neuromodulators of various neurotransmitter pathways to the brain, including those involved in depression (52). This in turn may have contributed to history of lung disease being a risk factor for depression as corroborated by Goodwin et al. (53).

Interestingly, the prevalence of depression is markedly higher in patients with NASH as compared with patients with simple hepatic steatosis. NASH with or without fibrosis is a more severe disease compared with simple steatosis. It occurs at a rate of about 25% in 3 years in patients in the simple steatosis stage (54). This would not come as a surprise given that an increase in circulating inflammatory cytokines is seen in steatohepatitis compared with simple steatosis, and patients with depression similarly see an increase in peripheral and central inflammation. In fact, the bidirectional relationship between depression and obesity, which is also closely related to NAFLD, has already been elucidated in animal studies (55). Therefore, it is likely that dietary initiatives protective toward NAFLD progression may also improve mental health in patients with depression (56). Evidently, depression and NAFLD share multiple upstream hits in the pathophysiology of the disease; thus, it is no wonder that they are gradiently associated.

In a study by Tomeno et al. the presence of depression was associated with a decreased effect of lifestyle intervention on weight loss, resulting in an increased challenge for clinical management (35). Although there are multiple pharmacotherapies with promising results in phase 3 trials for NASH, at present, weight loss and lifestyle change are the cornerstones of NAFLD treatment. Furthermore, overall weight loss has been shown to be beneficial for associated comorbidities such as diabetes and hypertension (57, 58). Yet, many antidepressant classes result in weight gain, and thus, the possibility of successful treatment outcome may be grim in the coexistence of depression and NAFLD (59).

It may be tempting to consider bariatric surgery for this subgroup of patients with both NAFLD and depression. After all, bariatric surgery has shown promising results in improvement in NASH as seen in several studies (60–62). Furthermore, weight loss following bariatric surgery may be associated with improvements in mood, at least initially. However, it is not without adverse outcomes. For example, a 10-year cohort study in Sweden reported an increase in suicides among obese patients following surgery compared with the general population (OR: 2.85, 95% CI: 2.40–3.39) in addition to alcohol and substance abuse (63). Several reasons for increased suicide rates post-bariatric surgery have been suggested, such as genetic susceptibility and changes in gut peptide release. It has also been theorized that addiction transfer could explain this phenomenon. In this theory, binge eating is hypothesized to serve as a coping mechanism to ameliorate negative emotional states (64). Consequently, in the post-bariatric group, this behavior is substituted by alcohol consumption due to the patient's inability to regulate emotions through overeating. However, this model has neither been adequately developed nor empirically validated and should be explored in future research (65).

Depression in NAFLD is associated with poorer outcomes such as decreased response to medical treatment and is even an independent predictor of all-cause mortality at 1 year. It is unclear if treatment of depression (be it pharmacological or behavioral) would positively influence NAFLD progression. Nevertheless, physicians should be cognizant of the fact that the prevalence and incidence of depression in NAFLD patients is higher and may consider adopting a low threshold in the use of screening scores for mood disorders in these patients as clinically required.

### Strengths and Limitations

To our best knowledge, this is the first meta-analysis of the prevalence, associative risk factors, and outcomes of depression in NAFLD patients. The limitation of this study is the heterogeneity in the included studies. However, we circumvented the issue by reclassification of depression diagnosis into clinician-rated, self-reported, and self-rated scales (66). Of note, the prevalence of depression was higher in self-reported and self-rated studies. Moreover, larger sample sizes are often associated with an increased *I*^2^ in simulation studies (67, 68). Thus, large *I*^2^ value (>90%) in prevalence meta-analysis may be attributed to the larger sample sizes involved (69, 70). Secondly, while the prevalence of NAFLD in Asia is similar to the Western population, only three out of the 10 studies were conducted in Asia. Thus, this meta-analysis may not adequately represent Asian NAFLD patients. Lastly, while our study reports factors associated with depression in NAFLD patients, more confirmatory studies are needed to show causation through further understanding of the pathogenesis and bidirectional relationship between the two conditions.

## Conclusion

In summary, this meta-analysis and systematic review assesses the association between depression and NAFLD. The analysis demonstrates the high prevalence of depression in NAFLD in spite of heterogeneity due to differences in diagnostic criteria of depression. Furthermore, patients with NASH were found to have a significantly higher risk of depression compared with those with NAFLD. Diabetes, BMI, history of smoking, history of lung disease, and being female were also identified as significant risk factors. NAFLD comorbid with depression has serious complications as evident in reduced clinical response after standard care and increased all-cause 1-year mortality. This paper, thus, highlights the significant clinical implications of NAFLD and depression as public health concerns. Further studies to validate the findings and explore potential pathophysiological mechanism underlying the association between depression and NAFLD are needed.

## Data Availability Statement

The original contributions presented in the study are included in the article/Supplementary Material, further inquiries can be directed to the corresponding authors.

## Author Contributions

JX, LL, and CN contributed to the acquisition of data, analysis and interpretation of data, and drafting of the article. DT, WL, CH, ET, AS, and MM aided in revising the article critically for important intellectual content. All authors read and gave final approval of the version to be submitted.

## Conflict of Interest

AS: President of Sanyal Biotechnology and has stock options in Genfit, Akarna, Tiziana, Indalo, Durect and Galmed. He has served as a consultant to Astra Zeneca, Nitto Denko, Enyo, Ardelyx, Conatus, Nimbus, Amarin, Salix, Tobira, Takeda, Jannsen, Gilead, Terns, Birdrock, Merck, Valeant, Boehringer-Ingelheim, Lilly, Hemoshear, Zafgen, Novartis, Novo Nordisk, Pfizer, Exhalenz and Genfit. He has been an unpaid consultant to Intercept, Echosens, Immuron, Galectin, Fractyl, Syntlogic, Affimune, Chemomab, Zydus, Nordic Bioscience, Albireo, Prosciento, Surrozen and Bristol Myers Squibb. His institution has received grant support from Gilead, Salix, Tobira, Bristol Myers, Shire, Intercept, Merck, Astra Zeneca, Malinckrodt, Cumberland and Novartis. He receives royalties from Elsevier and UptoDate. The remaining authors declare that the research was conducted in the absence of any commercial or financial relationships that could be construed as a potential conflict of interest.

## Abbreviations

BMI: body mass index
CES-D: Korean Center for Epidemiological Studies-Depression Scale
COPD: chronic obstructive pulmonary disease
DSM-IV-TR: Diagnostic and Statistical Manual of Mental Disorders, 4th edition, text revision
HADS: Hospital Anxiety and Depression Scale
ICD-9 and ICD-10: International Classification of Disease, Ninth Revision and Tenth Revision
NAFLD: non-alcoholic fatty liver disease
NASH: non-alcoholic steatohepatitis
MDD: major depressive disorder
PHQ-9: Patient Health Questionnaire-9.
