# Supplementary material for: Is Fatty Liver Associated With Depression? A Meta-Analysis and Systematic Review on the Prevalence, Risk Factors, and Outcomes of Depression and Non-alcoholic Fatty Liver Disease
Source: Front Med (Lausanne). 2021 Jun 30;8:691696. doi: 10.3389/fmed.2021.691696 (PMC8278401; doi:10.3389/fmed.2021.691696)
Supplement: Supplementary file 1 [file Table_1.DOCX]

Supplementary Material

# Supplementary Table 1: Summary of included studies

| **Study, Year** | **Country of origin** | **Study Design** | **Study Population: n; age mean (SD)** | **Depression (Events)** | **Summary of findings** |
| --- | --- | --- | --- | --- | --- |
| Elwing et al, 2006 | USA | Retrospective, case-controlled | 36; 51.4 (2) | 20 | Increased lifetime rates of Major Depressive Disorder (MDD) in NASH subjects compared with an obese comparison and after controlling for differences in diabetes rate and Waist-Hip Ratio (WHR) (OR: 3.8, CI: 1.4 – 10.2, p-value = 0.018) |
| Weinstein et al, 2011 | USA | Retrospective cohort | 184, 46.7 (11.2) | 50 | Increased prevalence of depression in patients with NAFLD in comparison with the general population. (NAFLD: 27.1 % vs general population: 2-5%) |
| Youssef et al, 2013 | USA | Retrospective cohort | 567; 48 (11) | 80 | Patients with more severe depressive symptom were associated with higher likelihood of having more severe hepatocyte ballooning.  Cumulative odds ratio (COR) of subclinical and  clinical depression for having a higher grade of hepatocyte ballooning were  2.1 (CI: 1.0 - 4.4) and 3.6 (CI: 1.4 - 8.8) respectively. |
| Tomeno et al, 2016 | Japan | Prospective cohort | 258; 50.2 (14.14) | 32 | NAFLD patients comorbid with MDD have more severe histological steatosis grade (p-value = 0.0034), higher levels of serum AST (73.1 and 51.0, p-value = 0.0044), serum ALT (105.2 and 79.3, p-value = 0.0128), GGT (150.0 and 78.0, p-value = 0.0007), ferritin (339.7 mg/ml and 233.5 mg/ml, p-value = 0.0089), and  hs-CRP (0.327 mg/dl and 0.164 mg.dl, p-value = 0.0040) than NAFLD patients without MDD.  Moreover, NAFLD patients comorbid with MDD had less reactivity to the 48 weeks standard care, provided mainly by lifestyle modification, compared to NAFLD without MDD. |
| Balp et al, 2019 | Germany, France, Spain | Retrospective cohort | 184; 54.5 (13.1) | 57 | Patients with NASH reported significantly higher prevalence of depression than the general population (NASH: 31.20% and general population: 19.60%, p-value = 0.001) |
| Jung et al, 2019 | Korea | Retrospective cohort | 31635; 41.81 (7.425) | 2870 | Depression was significantly associated with more advanced condition of NAFLD. Adjusted OR of depression for mild fatty liver is 1.14 (CI: 1.06 – 1.22) and for moderate to severe fatty liver, adjusted OR is 1.39 (CI: 1.17 – 1.48). |
| Fernández et al, 2020 | Cuba | Retrospective cohort | 221; 54 (11.3) | 86 | High prevalence of depression in patients with NAFLD, differences in prevalence of depression between different etiology of chronic liver disease was not statistically significant (p-value = 0.08) |
| Labenz et al, 2020 | Germany | Retrospective cohort | 19871; 58.5 (14.2) | 4213 | NAFLD is associated with development of depression compared to matched controls without NAFLD, (HR: 1.21, CI: 1.14 – 1.26, p-value <0.001). |
| Sayiner et al, 2020 | USA | Retrospective cohort | 1980950; 70.11 (11.13) | 188307 | Prevalence of depression in NAFLD patients is 5.84%  Odds ratio for 1-year all-cause mortality in NAFLD patients comorbid with depression is 1.07 (CI: 1.05 – 1.09) in inpatient setting and 1.21 (CI: 1.18 – 1.25) in outpatient setting. |
| Choi et al,  2021 | Korea | Retrospective cross-sectional | 7846; 49.40  (10.04) | 335 | Presence of NAFLD had no association with depression (risk of depression in NAFLD vs control, OR: 1.09, CI: 0.95 – 1.26). |

# Supplementary Table 2: Risk of Bias Assessment

| Study | 1 | 2 | 3 | 4 | 5 | 6 | 7 | 8 | 9 | Risk of bias |
| --- | --- | --- | --- | --- | --- | --- | --- | --- | --- | --- |
| Elwing et al, 2006 | Y | Y | N | Y | N | Y | Y | Y | N | Low |
| Weinstein et al, 2011 | Y | Y | Y | Y | Y | N | Y | Y | Y | Low |
| Youssef et al, 2013 | Y | Y | Y | Y | Y | N | Y | Y | Y | Low |
| Tomeno et al, 2015 | Y | Y | Y | N | Y | Y | Y | N | Y | Low |
| Balp et al, 2019 | Y | N | Y | Y | N | N | Y | Y | N | Moderate |
| Jung et al, 2019 | Y | Y | Y | Y | Y | N | Y | Y | N | Low |
| Fernández et al, 2020 | Y | Y | Y | Y | Y | N | U | Y | Y | Low |
| Labenz et al, 2020 | Y | N | Y | N | N | Y | Y | Y | Y | Moderate |
| Saviner et al, 2020 | Y | Y | Y | Y | N | Y | U | Y | N.A. | Moderate |
| Choi et al, 2021 | Y | Y | Y | Y | Y | N | Y | Y | U | Low |

U - Unclear

N.A. – Not Applicable

**JBI Critical Appraisal Checklist**

1. Was the sample frame appropriate to address the target population?

2. Were study participants sampled in an appropriate way?

3. Was the sample size adequate?

4. Were the study subjects and the setting described in detail?

5. Was the data analysis conducted with sufficient coverage of the identified sample?

6. Were valid methods used for the identification of the condition?

7. Was the condition measured in a standard, reliable way for all participants?

8. Was there appropriate statistical analysis?

9. Was the response rate adequate, and if not, was the low response rate managed appropriately?

**3 Supplementary Table 3: PRISMA 2009 Checklist**

| **Section/topic** | **#** | **Checklist item** | **Reported on page #** |
| --- | --- | --- | --- |
| **TITLE** | | |  |
| Title | 1 | Identify the report as a systematic review, meta-analysis, or both. | 1 |
| **ABSTRACT** | | |  |
| Structured summary | 2 | Provide a structured summary including, as applicable: background; objectives; data sources; study eligibility criteria, participants, and interventions; study appraisal and synthesis methods; results; limitations; conclusions and implications of key findings; systematic review registration number. | 3-4 |
| **INTRODUCTION** | | |  |
| Rationale | 3 | Describe the rationale for the review in the context of what is already known. | 5 |
| Objectives | 4 | Provide an explicit statement of questions being addressed with reference to participants, interventions, comparisons, outcomes, and study design (PICOS). | 5 |
| **METHODS** | | |  |
| Protocol and registration | 5 | Indicate if a review protocol exists, if and where it can be accessed (e.g., Web address), and, if available, provide registration information including registration number. | 6 |
| Eligibility criteria | 6 | Specify study characteristics (e.g., PICOS, length of follow-up) and report characteristics (e.g., years considered, language, publication status) used as criteria for eligibility, giving rationale. | 6 |
| Information sources | 7 | Describe all information sources (e.g., databases with dates of coverage, contact with study authors to identify additional studies) in the search and date last searched. | 6 |
| Search | 8 | Present full electronic search strategy for at least one database, including any limits used, such that it could be repeated. | 6 |
| Study selection | 9 | State the process for selecting studies (i.e., screening, eligibility, included in systematic review, and, if applicable, included in the meta-analysis). | 6 |
| Data collection process | 10 | Describe method of data extraction from reports (e.g., piloted forms, independently, in duplicate) and any processes for obtaining and confirming data from investigators. | 6-7 |
| Data items | 11 | List and define all variables for which data were sought (e.g., PICOS, funding sources) and any assumptions and simplifications made. | 7 |
| Risk of bias in individual studies | 12 | Describe methods used for assessing risk of bias of individual studies (including specification of whether this was done at the study or outcome level), and how this information is to be used in any data synthesis. | 8 |
| Summary measures | 13 | State the principal summary measures (e.g., risk ratio, difference in means). | 8 |
| Synthesis of results | 14 | Describe the methods of handling data and combining results of studies, if done, including measures of consistency (e.g., I^2^) for each meta-analysis. | 8 |

| **Section/topic** | **#** | **Checklist item** | **Reported on page #** |
| --- | --- | --- | --- |
| Risk of bias across studies | 15 | Specify any assessment of risk of bias that may affect the cumulative evidence (e.g., publication bias, selective reporting within studies). | N.A. |
| Additional analyses | 16 | Describe methods of additional analyses (e.g., sensitivity or subgroup analyses, meta-regression), if done, indicating which were pre-specified. | 10 |
| **RESULTS** | | |  |
| Study selection | 17 | Give numbers of studies screened, assessed for eligibility, and included in the review, with reasons for exclusions at each stage, ideally with a flow diagram. | 9 |
| Study characteristics | 18 | For each study, present characteristics for which data were extracted (e.g., study size, PICOS, follow-up period) and provide the citations. | 9 |
| Risk of bias within studies | 19 | Present data on risk of bias of each study and, if available, any outcome level assessment (see item 12). | Supplementary material: Table 2 |
| Results of individual studies | 20 | For all outcomes considered (benefits or harms), present, for each study: (a) simple summary data for each intervention group (b) effect estimates and confidence intervals, ideally with a forest plot. | 9-13 |
| Synthesis of results | 21 | Present results of each meta-analysis done, including confidence intervals and measures of consistency. | 9-13 |
| Risk of bias across studies | 22 | Present results of any assessment of risk of bias across studies (see Item 15). | N.A. |
| Additional analysis | 23 | Give results of additional analyses, if done (e.g., sensitivity or subgroup analyses, meta-regression [see Item 16]). | 10 |
| **DISCUSSION** | | |  |
| Summary of evidence | 24 | Summarize the main findings including the strength of evidence for each main outcome; consider their relevance to key groups (e.g., healthcare providers, users, and policy makers). | 14 |
| Limitations | 25 | Discuss limitations at study and outcome level (e.g., risk of bias), and at review-level (e.g., incomplete retrieval of identified research, reporting bias). | 15-16 |
| Conclusions | 26 | Provide a general interpretation of the results in the context of other evidence, and implications for future research. | 16 |
| **FUNDING** | | |  |
| Funding | 27 | Describe sources of funding for the systematic review and other support (e.g., supply of data); role of funders for the systematic review. |  |
